# Post-sepsis psychiatric disorder: Pathophysiology, prevention, and treatment

**DOI:** 10.1007/s10072-024-07409-8

**Published:** 2024-02-21

**Authors:** Dayong Li, Xujie Zhang, Yuru Lu, Liang Jing, Hongjie Hu, Yaqin Song, Shuhui Wu, Wei Zhu

**Affiliations:** 1 Department of Emergency Medicine, Tongji Hospital, Tongji Medical College, Huazhong University of Science and Technology, Wuhan, China; 2 Department of Intensive Care Medicine, Tongji Hospital, Tongji Medical College, Huazhong University of Science and Technology, 1095 Jiefang Ave, Wuhan, 430030 Hubei China

**Keywords:** Sepsis, Psychiatric disorder, Pathophysiology, Prevention, Therapy

## Abstract

Post-sepsis psychiatric disorder, encompassing anxiety, depression, post-traumatic stress disorder and delirium, is a highly prevalent complication secondary to sepsis, resulting in a marked increase in long-term mortality among affected patients. Regrettably, psychiatric impairment associated with sepsis is frequently disregarded by clinicians. This review aims to summarize recent advancements in the understanding of the pathophysiology, prevention, and treatment of post-sepsis mental disorder, including coronavirus disease 2019-related psychiatric impairment. The pathophysiology of post-sepsis psychiatric disorder is complex and is known to involve blood–brain barrier disruption, overactivation of the hypothalamic–pituitary–adrenal axis, neuroinflammation, oxidative stress, neurotransmitter dysfunction, programmed cell death, and impaired neuroplasticity. No unified diagnostic criteria for this disorder are currently available; however, screening scales are often applied in its assessment. Modifiable risk factors for psychiatric impairment post-sepsis include the number of experienced traumatic memories, the length of ICU stay, level of albumin, the use of vasopressors or inotropes, daily activity function after sepsis, and the cumulative dose of dobutamine. To contribute to the prevention of post-sepsis psychiatric disorder, it may be beneficial to implement targeted interventions for these modifiable risk factors. Specific therapies for this condition remain scarce. Nevertheless, non-pharmacological approaches, such as comprehensive nursing care, may provide a promising avenue for treating psychiatric disorder following sepsis. In addition, although several therapeutic drugs have shown preliminary efficacy in animal models, further confirmation of their potential is required through follow-up clinical studies.

## Introduction

Sepsis is a major threat to human health worldwide. In 2017, there were an estimated 48.9 million cases of sepsis globally, resulting in approximately 11 million deaths, representing 19.7% of the total [1]. As knowledge of the pathogenesis of this condition has increased, treatments have become more effective, resulting in a steady decrease in sepsis-related in-hospital mortality over time [2]. However, many sepsis survivors experience cognitive, psychiatric, and physical impairment, collectively known as post-sepsis syndrome, which has emerged as a key area of concern [3]. The consequences of sepsis are long-lasting, and, although often overlooked, can lead to substantial morbidity and mortality [4]. By actively exploring the pathogenesis and strategies for the prevention of post-sepsis syndrome, the incidence of these disorders can be effectively decreased, and the health status and quality of life of sepsis survivors can be improved.

Typically, the psychiatric disorder of sepsis survivors manifests as depression, anxiety, and post-traumatic stress disorder (PTSD), all of which can have a profound influence on their daily life as well as their ability to return to work following recovery [5, 6]. Additionally, delirium is also a neuropsychiatric symptom that can occur after sepsis. It manifests in a complex form, characterized by cognitive deficits, attention deficits, circadian dysrhythmia, emotional dysregulation, and alteration in psychomotor function [7]. Over the last decade, many studies have focused on investigating cognitive dysfunction and functional disability following sepsis [8]. In comparison, relatively few studies have concentrated on the psychiatric impairment that can arise secondary to sepsis. Hence, the aim of this study was to systematically review current knowledge regarding the pathogenesis, prevention, and treatment of post-sepsis psychiatric disorder, including depression, anxiety, PTSD and delirium.

## Epidemiology

A study from Germany reported that sepsis survivors experience a high prevalence of psychiatric disorder in the first three years after discharge, with rates of 43.7%, 47.8%, and 48.5% in the first, second, and third years, respectively. The new onset of psychological impairment in the same period was respectively 17.9%, 12.8%, and 12.2% [9]. Delirium is a frequently observed complication in ICU patients with sepsis, with an incidence ranging from 17.7% to 48% [10, 11]. A retrospective cohort study revealed that patients with sepsis-associated delirium (SAD) had a higher mortality rate at 28 days and longer ICU stays compared to patients without SAD [12]. Moreover, individuals with SAD had a prolonged duration of mechanical ventilation and a significantly higher likelihood of needing professional care post-discharge [10]. Depression, anxiety, and PTSD often occur simultaneously, impacting overall health-related quality of life (HRQOL) [13]. One study reported incidences of anxiety, depression, and PTSD of respectively 67%, 49%, and 46% among sepsis survivors 24 h after discharge from the ICU. In comparison, patients who are re-evaluated one year after ICU discharge show incidence rates of respectively 38%, 50%, and 31%. The incidence of anxiety and PTSD decreases over time, whereas that of depression exhibits a significant increase [14]. The presence of persistent depressive symptoms has been found to strongly correlate with increased long-term mortality. This is supported by a large cohort study conducted in the UK, in which it was found that depressive symptoms are associated with an increased risk of mortality two years after discharge from the ICU [13]. A different study revealed that patients with post-sepsis depression had a five-year all-cause mortality rate of 46.2%, which was 1.29-fold higher than that of control patients. The same study also found that post-sepsis depression is independently linked to a higher five-year all-cause mortality among sepsis survivors [15]. To better understand the progression of depression following sepsis, Boede et al. identified three distinct trajectories of depressive symptoms one year after ICU discharge, namely, mild recovery, severe persistence, and severe recovery. Surprisingly, over one-third of patients did not recover from depression. Furthermore, the severity and duration of depression were found to be linked with chronic pain, PTSD, and a decrease in HRQOL [16]. These observations highlight the need to decrease the prevalence of post-sepsis psychiatric disorder among affected patients and enhance their quality of life.

## Pathophysiology

### Blood–brain barrier

The blood–brain barrier (BBB) is responsible for regulating the entry of nutrients and immune cells into the brain, while also excluding toxic substances. This crucial function helps maintain the homeostasis of the central nervous system [17]. The BBB is mainly composed of specialized endothelial cells, glycocalyx, and a basement membrane that includes pericytes and astrocyte end-feet [18]. Cerebrovascular endothelial cells are the key components of the BBB and are responsible for regulating the permeability of the BBB through tight junctions [17]. There is evidence supporting that the disruption of BBB structure may be associated with psychiatric disorder following sepsis. One study found that mice display anxiety and depression-like behaviors one day after cecal ligation and puncture (CLP) surgery, which mimics human sepsis, accompanied by a marked aggregation of endothelial cell nuclear chromatin at the edge of the nuclear membrane. Additionally, a significant increase in the permeability of the BBB to Evans blue was observed in the mouse brain, indicative of BBB disruption [19]. However, the exact cause of this effect is not clear but may involve astrocyte activation. Studies have demonstrated that astrocytes are activated during sepsis and release inflammatory mediators, such as interleukin-1 beta (IL-1β) and IL-6, which then disrupt the integrity of the BBB during the early phase post-CLP, thereby further promoting psychiatric impairment [20, 21].

### Neuroinflammation

Systemic inflammation during sepsis leads to the production of proinflammatory cytokines. Peripheral proinflammatory cytokines, such as IL-1β and tumor necrosis factor-alpha (TNF-α), can enter the brain parenchyma by binding to receptors on brain endothelial cells, and subsequently activate glial cells and induce neuroinflammation, resulting in a vicious circle [22–24]. Evidence suggests that there may be a correlation between neuroinflammation and psychiatric symptoms following sepsis. Indeed, mice display anxiety-like behavior 10 days after CLP, along with increased levels of TNF-α, interferon-gamma (IFN-γ), IL-1β, and IL-6 in brain tissue [25]. Additionally, the inhibition of the nuclear factor-kappa B (NF-κB) pathway can reduce microglial activation, thereby alleviating psychiatric symptoms in septic mice [26]. Nevertheless, the precise mechanism underlying how neuroinflammation leads to post-sepsis psychiatric disorder is incompletely understood. We have previously found that the activation of the NOD-like receptor pyrin domain-containing protein 3 (NLRP3) inflammasome induces a notable rise in the levels of IL-1β, IL-18, and TNF-α in the hippocampus of septic mice and promotes the activation of microglia [27], which can then further activate the NLRP3 inflammasome [28]. Activated microglia also promote the production and release of a significant number of inflammatory mediators, which facilitate the expression of indoleamine 2,3-dioxygenase (IDO), a key enzyme in endotoxin-induced depression [29]. Heightened IDO activity not only results in tryptophan depletion but also triggers the production of neurotoxic products such as 3-hydroxytryptophan and quinolinic acid, which jointly contribute to the development of depression [30].

### Oxidative stress

Oxidative stress and neuroinflammation are interrelated and can exacerbate each other. Oxidative stress occurs when there is an overproduction of reactive oxygen species (ROS) and an inadequate capacity for scavenging these molecules through the antioxidant system [31]. Studies have confirmed that neuroinflammation triggers the release of proinflammatory mediators, which subsequently activate microglia and promote the production of ROS, ultimately leading to neuronal damage. Oxidative stress also contributes to the propagation of inflammatory signaling pathways [32]. Emerging evidence supports that oxidative stress plays a role in the development of psychiatric disorder following sepsis. For instance, mice exhibit anxiety- and depression-like behaviors one month after intraperitoneal injection of lipopolysaccharide (LPS), concomitant with an increase in the expression of the inducible nitric oxide synthase (iNos) gene and oxidative stress markers in the hippocampus and prefrontal cortex [33]. Similarly, rats display marked anxiolytic behavior following CLP, accompanied by an increase in malondialdehyde content and a significant decrease in superoxide dismutase activity [34]. The mechanisms involved in how oxidative stress contributes to post-sepsis psychiatric disorder are not clear. Nevertheless, as oxidative stress is closely related to neuroinflammation, it is likely that a close relationship exists between oxidative stress and neuroinflammation in the occurrence of post-sepsis psychiatric disorder.

### Programmed cell death

Programmed cell death (PCD) comprises apoptosis, cuproptosis, pyroptosis, autophagy, necroptosis, and ferroptosis [35]. An increasing number of studies have suggested that PCD is involved in the occurrence of psychiatric impairment secondary to sepsis. Mice exhibit depressive behavior 14 days after CLP, which is accompanied by increased neuronal apoptosis and necroptosis in the hippocampus [36]. Additionally, the expression of myelocyte differentiation factor 2 (MD2), an intermediary regulator of apoptosis and necroptosis [37], is significantly increased in hippocampal neurons 24 h after CLP. Compared with wild-type mice, transgenic mice exhibiting excitatory neuron-specific MD2 knockout display less depression-like behavior two weeks following CLP, along with a reduction in the expression of apoptosis- and necroptosis-related proteins, as well as fewer dead neurons in the hippocampus. These findings indicate that MD2-mediated apoptosis and necroptosis are associated with the onset of post-sepsis depression [36]. Another study reported that mice show obvious anxiety- and depression-like behaviors on days 1 and 7 post-CLP concurrently with an increase in the levels of pyroptosis-related proteins in the cerebral cortex. However, this increase is blocked following the administration of a caspase-1 inhibitor, ultimately leading to an improvement in anxiolytic and depressive behaviors in the animals [19]. These findings implicate pyroptosis in the onset of psychiatric symptoms after sepsis.

### Neuroplasticity

There is evidence to suggest that impaired neuroplasticity, a process involving neurogenesis, axon sprouting, axon regeneration, and synaptic plasticity [38], is linked to the occurrence of psychiatric disorder following sepsis. Mice display anxiety- and depression-like behaviors one month after LPS injection, along with a notable decrease in the expression of activity-regulated cytoskeletal-associated protein (ARC) and early growth response 1 (EGR1), synaptic plasticity-related early gene products. There is also a marked reduction in neural stem cell proliferation in the subgranular zone of the dentate gyrus in these animals [26]. The reduction in the expression of synaptic proteins and the elimination of synapses is indicative of impaired synaptic plasticity. One study revealed that mice exhibiting noticeable signs of psychiatric disorder after CLP also display a decrease in the expression levels of the presynaptic protein synapsin 1 and the postsynaptic proteins *N*-methyl-*D*-aspartate receptor subunit 2B (NR2B) and postsynaptic density protein 95 (PSD-95) in the hippocampus and cortex. However, treatment with a caspase-1 inhibitor recovered the levels of above-mentioned proteins [19]. The C1q complement pathway is thought to mediate synaptic elimination. A recent study demonstrated that translocator protein (Tspo)-knockout mice display more severe psychiatric symptoms 17 days after CLP, together with an upregulation of C1q complement pathway-related genes in the hippocampus [39]. These observations indicate that post-sepsis psychiatric disorder is associated with synaptic elimination mediated by the C1q complement pathway. Brain-derived neurotrophic factor (BDNF) and its related signaling pathways have been confirmed to play a crucial role in neuroplasticity. Rats exhibit evident depressive behavior 10 days post CLP, concomitant with a reduction in BDNF levels in the hippocampus [40], indirectly suggesting that neuroplasticity is associated with psychiatric impairment secondary to sepsis.

### The hypothalamic–pituitary–adrenal axis

The hypothalamic–pituitary–adrenal (HPA) axis is an important component of the neuroendocrine system. Under conditions of stress, neurons in the hypothalamic paraventricular nucleus (PVN) synthesize and release corticotropin-releasing hormone (CRH), which then binds to CRH1 and CRH2 receptors in the anterior pituitary, thus inducing the release of adrenocorticotropic hormone (ACTH) into the bloodstream. ACTH promotes the secretion of glucocorticoid (GC) from the adrenal gland. GC, in turn, binds to the glucocorticoid receptor (GR) in the hippocampus, PVN, and anterior pituitary, thus creating a negative feedback loop that inhibits the release of CRH and contributes to the maintenance of body homeostasis [41]. Studies have shown that overactivation of the HPA axis is associated with systemic inflammation-induced anxiety and depression [42, 43], suggesting that it may also be involved in post-sepsis psychiatric disorder. In addition to depression-like behavior, sepsis survivor rats exhibit increased adrenal weight and corticosterone and ACTH contents [40], suggesting that activation of the HPA axis is involved in the onset of depression after sepsis. A subsequent study showed that the heightened reactivity of the HPA axis may be attributed to the dysfunction of the ventral hippocampus [44].

### Neurotransmitters

Neurotransmitter abnormalities are closely related to the occurrence of neuropsychiatric disorders. Neurotransmitters such as gamma amino butyric acid (GABA), dopamine, norepinephrine, glutamate, and acetylcholine have been reported to be abnormally expressed during sepsis [45], suggesting that they may be involved in post-sepsis psychiatric disorder. One study noted that the anxiety-like behavior seen in rats after CLP was correlated with the activation of the IL-1β/GABA type A receptor (GABAAR) pathway in the hippocampus [34]. Meanwhile, a different study found that post-sepsis anxiety is associated with a decrease in the expression of 5-hydroxytryptamine 1A receptor (5-HT1AR) and an increase in that of 5-HT2AR [46]. Glutamate is an excitatory neurotransmitter. Enhanced intracellular system x_c_^−^ activity leads to the release of glutamate from activated microglia, which contributes to the depression-like behavior seen in septic mice after the intraperitoneal injection of LPS. Interestingly, the use of the glutamate receptor antagonists MK801 and DNQX alleviated these depressive symptoms [47], indicating that a link may exist between glutamate and the occurrence of depression following sepsis. During neuroinflammation, IDO converts tryptophan, the precursor of serotonin, into quinolinic acid, which can bind to the *N*-methyl-*D*-aspartate (NMDA) receptor and exert excitatory neurotoxic effects [48]. It has been observed that the heightened expression of IDO in the hippocampus and prefrontal cortex is related to psychiatric symptoms following LPS injection [33]. In summary, the disruption of the neurotransmitter system promotes the occurrence of psychiatric disorder following sepsis.

## Diagnosis

Given the lack of uniform diagnostic criteria for psychiatric disorder after sepsis, screening scales are frequently used to assess the occurrence of anxiety, depression, delirium and PTSD in sepsis survivors. The Beck Anxiety Inventory (BAI) and the Self-rating Anxiety Scale (SAS) are commonly utilized for the self-assessment of anxiety, while the Major Depression Inventory (MDI), Beck Depression Inventory-II (BDI-II), Self-rating Depression Scale (SDS), and Patient Health Questionnaire-9 (PHQ-9) are widely employed for the self-assessment of depression. In hospital settings, the Hospital Anxiety and Depression Scale (HADS) is often used for the screening of patients for anxiety and depression. The Confusion Assessment Method for the Intensive Care Unit (CAM-ICU) and the Intensive Care Delirium Screening Checklist (ICDSC) are widely used assessment tools for detecting delirium in intensive care unit patients. Self-assessment tools such as the PTSD checklist for DSM-5 (PCL-5), Posttraumatic Symptom Scale-10 (PTSS-10), and Impact of Events Scale-Revised (IES-R) are normally used to evaluate the symptoms of PTSD. However, for a formal diagnosis, professionals rely on the more complex and comprehensive structured interview Clinician-administered PTSD Scale for DSM-5 (CAPS-5), which is considered the gold standard in PTSD diagnosis. A detailed summary of these scales is provided in Table 1. In 2019, the Society of Critical Care Medicine (SCCM) of the United States issued a consensus statement regarding the prediction and identification of post-intensive care syndrome [49]. In this statement, HADS was highly recommended for assessing anxiety and depression, with a score of 8 or higher indicating significant anxiety or depression. For assessing PTSD, IES-R and IES-6 were weakly recommended, with respective optimal cut-off values of 1.6 and 1.75. These suggestions could also serve as a reference for identifying post-sepsis psychiatric disorder.

**Table 1:** Screening scales for post-sepsis psychiatric disorder

Scale	No. of items	Total score	Score interpretation	Administration	Strength	Limitation	Reference
Anxiety							
BAI	21	63	0–9 normal, 10–18 mild to moderate, 19–29 moderate to severe, 30–63 severe	Self-report	Brief, easy to use, sound psychometric properties	Limited scope with a focus on somatic symptoms; not free	[14, 50]
HADS-A	7	21	0–7 normal, 8–10 mild, 11–14 moderate, 15–21 severe	Self-report	Brief, widely used, easily obtained	Reduced validity in some populations (e.g. the aged); inability to specifically screen anxiety disorder	[50–52]
SAS	20	80 (raw score); 100 (index score)	Index score: 45–59 mild to moderate, 60–74 moderate to severe, 75–100 severe	Self-report	Widely used, high sensitivity	Easy to confuse the index score with the raw score, resulting in an increase in the cut-off value for significance	[53–55]
Depression							
MDI	10	50	Moderate depression: a score ≥ 4 for two of the three top items and at least four of the remaining items; major depression: a score ≥ 4 on five of nine items (excluding item 4), and one of these five items should be depressed mood or loss of interest	Self-report	Brief, valid	Relies on the cooperation and reading ability of patients	[56–59]
BDI-II	21	63	0–13 minimal, 14–19 mild, 20–28 moderate, 29–63 severe	Self-report	Fast, widely used, psychometric properties	Depressive symptoms easily overlap with medical conditions; not free	[14, 60]
SDS	20	80 (raw score); 100 (index score)	Index score: 25–49 normal, 50–59 mild to moderate, 60–69 moderate to severe, 70–100 severe	Self-report	Widely used, high sensitivity	Easy to confuse the index score with the raw score	[53, 55]
HADS-D	7	21	0–7 normal, 8–10 mild, 11–14 moderate, 15–21 severe	Self-report	Fast, brief, widely used	Narrow scope, with a focus on cognitive and emotional aspects of depression	[51, 52, 60]
PHQ-9	9	27	1–4 normal, 5–9 mild, 10–14 moderate, 15–19 moderately severe, 20–27 severe	Self-report	Fast, strong psychometric properties, widely used, free	Low specificity	[16, 60–62]
PTSD							
PCL- 5	20	80	A score of 33 serves as a cut-off for a provisional PTSD diagnosis	Self-report	Widely used, strong psychometric properties, fast	Only evaluates PTSD symptoms, and does not assess trauma-related symptoms	[63–66]
PTSS-10	10	70	A score above 35 indicates clinically relevant PTSD	Self-report	Widely used, high specificity and sensitivity	Developed according to DSM-III rather than DSM-V diagnostic criteria, may not be appropriate for current use	[16, 66–68]
CAPS-5	30	80	The higher the score the higher the PTSD symptom severity	Structured clinical interview	Gold standard, excellent psychometric properties	Slow; requires additional training for standard administration	[64–66, 69]
IES-R	22	88	A score ≥ 33 indicates a high risk of PTSD	Self-report	Short, easy to use, different copies are available	Not fully aligned with DSM-V diagnostic criteria	[64, 67, 70, 71]
Delirium							
CAM-ICU	4	-	Positive for delirium: item 1 and 2 plus either item 3 or 4	Observation and interaction at one time point	Widely used, high specificity and sensitivity	Requiring patients’ cooperation, not convenient for ICU nurses	[72]
ICDSC	8	8	Total score ≥ 4 indicates the presence of delirium	Continuous observation of routine care	High sensitivity, easy to use	Low specificity	[72]

## Prevention

Psychiatric disorder following sepsis significantly impacts the daily life and long-term prognosis of sepsis survivors. Thus, implementing targeted preventive measures to reduce the incidence of related psychiatric disorder is of great clinical significance. According to the consensus statement issued by the SCCM, individuals who experience anxiety, depression, and PTSD before the onset of severe illness; acquire fear memories in the ICU; and lack social support during their illness are at a higher risk for long-term psychiatric impairment after critical illness. Accordingly, it is recommended that these patients undergo early screening and evaluation for psychiatric problems after discharge, as well as following important changes in health or life status [49].

The precipitating factors for post-sepsis psychiatric disorder may be multifactorial. In a prospective cohort study involving 135 patients with abdominal sepsis, 28% of participants reported moderate PTSD symptom scores while 10% reported high PTSD symptom scores one year after laparotomy. Correlations were observed between the occurrence of PTSD and factors such as age, length of stay in the ICU, and traumatic memories during hospitalization. After multivariate adjustment, the acquisition of more than four traumatic memories during hospitalization and longer stay in the ICU were determined to be independent risk factors for PTSD [67]. A prospective cohort study conducted on 439 elderly survivors of severe sepsis reported that 28% of patients experienced depressive symptoms following sepsis. Interestingly, clinical characteristics such as organ dysfunction score, length of hospital stay, and mechanical ventilation were not found to be associated with subsequent depression. However, suffering from depression before sepsis and functional impairment after sepsis were identified as independent risk factors for post-sepsis depression [73]. In another single-center study involving 33 patients with severe sepsis or septic shock, a positive association was identified between serum S100β levels and the incidence of PTSD. The cumulative dose of dobutamine during ICU stay was also positively correlated with the development of depression [14]. According to a study conducted in Korea, several risk factors for SAD were identified. These include older age (≥ 65), dependency in activities, high care needs, low awareness, shortness of breath, and thrombocytopenia. On the other hand, high level of albumin and the use of vasopressors or inotropes were found to potentially reduce the risk of SAD. Moreover, the risk of delirium was observed to increase with the severity of organ dysfunction [10]. The studies presented above suggest that there are several modifiable risk factors for psychiatric impairment after sepsis, including the number of traumatic memories, length of stay in the ICU, level of albumin, the use of vasopressors or inotropes, daily activity function after sepsis, and the cumulative dose of dobutamine. Primary care management includes evidence-based, post-sepsis care training for patients and their primary caregivers, case management by trained nurses, and clinical decision support provided by consulting physicians. It has been observed that, compared with usual care, primary care management can effectively reduce the incidence of PTSD among sepsis survivors one year after intervention [74]. Furthermore, in a prospective multicenter cohort study involving patients with sepsis, it was found that complete performance of the ABCDEF (Assess, prevent, and manage pain; Both spontaneous awakening and breathing trials; Choice of Analgesia and Sedation; Delirium assess, prevent, and manage; Early Mobility and Exercise; Family engagement/empowerment) bundle was associated with a reduced likelihood of delirium [75].

In summary, interventions targeting these modifiable risk factors, coupled with primary care management and the ABCDEF bundle, potentially serve as an effective strategy for the prevention of post-sepsis psychiatric disorder. However, there is still a lack of relevant research on prevention, which necessitates the further implementation of high-quality studies.

## Treatment

No established treatment for post-sepsis psychiatric disorder is currently available. Research in this area is still in its infancy. Potential effective treatment options include both pharmacological and non-pharmacological therapies.

### Non-pharmacological therapy

Internet-based cognitive behavioral therapy (ICBT) is a web-based writing intervention that is accessible and spatially independent. The treatment encompasses three aspects, namely, resource-oriented biographical reconstruction, in sensu trauma exposure, and cognitive reconstruction. After each writing session, a professional psychotherapist provides timely feedback. However, the therapeutic value of ICBT is inconclusive. For instance, in one case report, ICBT ameliorated PTSD symptoms in a sepsis survivor and his spouse [69], whereas in a different study, this intervention was reported to be ineffective at treating PTSD symptoms after severe sepsis. However, the small sample size in the latter study may have contributed to the negative result [63]. More studies with larger sample sizes are required to determine the efficacy of ICBT. Comprehensive nursing care is a widely utilized approach in the clinical care of surgical patients and individuals with chronic diseases. To ensure high-quality nursing care, all aspects of nursing work should be comprehensively carried out following established nursing procedures. Comprehensive nursing care is effective at reducing the levels of anxiety and depression in sepsis patients, leading to improved prognosis and quality of life [53]. In addition, it has demonstrated that early physical and occupational therapy can effectively decrease the duration of delirium in critically ill patients who require mechanical ventilation, including those with sepsis [76].

### Pharmacological therapy

CLP and intraperitoneal injection of LPS are the most commonly used strategies for generating animal models of sepsis for the investigation of psychiatric disorder secondary to sepsis. Several drugs, such as imipramine, dexamethasone, guanosine, and nicotine, are effective therapies in animal models. These drugs exert their effects through a variety of mechanisms, including improving the neuroinflammatory response, reducing oxidative stress, inhibiting HPA axis activation, and decreasing BBB permeability (see Table 2 for details). Recent years have seen rapid development in cell therapy, particularly in the use of mesenchymal stem cells, with studies having shown that these cells possess anti-inflammatory and neuroprotective properties [77]. Mesenchymal stem cells and their conditioned medium can alleviate anxiety-like behavior in rats following CLP, effects that are achieved through the alleviation of the inflammatory response and astrocyte activation [20, 46]. These preliminary findings provide promising prospects for the management of psychiatric conditions following sepsis.

**Table 2:** Promising drugs and their mechanisms of action

Study	Treatment	Model, Strain	Domain	Mechanism	Target
Xu et al. [19]	VX765 (0.2 mg, intragastric)	CLP, BALB/c mice	Anxiety and depression↓	Pyroptosis↓, BBB disruption↓, inflammatory cytokine levels↓, microglia activation↓, synaptic plasticity↑	Caspase-1
Silva et al. [20]	Mesenchymal stromal cells (1 × 10^5^ cells, iv)	CLP, Swiss Webster mice	Anxiety↓	BBB dysfunction↓, astrocyte activation↓, levels of inflammatory mediators↓	–
Anderson et al. [26]	PDTC (200 mg/kg, ip)	LPS, C57BL/6 mice	Anxiety and depression↓	Microglia activation↓, EGR1↑	NF-κB
Fang et al. [28]	Indole-3-propionic acid (25 mg/kg, gavage)	CLP, specific-pathogen-free C57BL/6 mice	Anxiety↓	NLRP3 inflammasome↓	Aryl hydrocarbon receptor
Casaril et al. [33]	CMI (1 mg/kg, oral)	LPS, Swiss mice	Anxiety and depression↓	Neutrophil numbers↓, reactive oxygen species↓, BBB dysfunction↓, inflammation-associated genes↓, oxidative stress markers↓	–
Ranjbaran et al. [34]	Tannic acid (20 mg/kg, ip)	CLP, Wistar rats	Anxiety↓	Inflammatory markers↓, oxidative stress↓	IL-1β/GABAAR
Fan et al. [36]	Tat-CIRP (50 mg/kg, ip)	CLP, C57/BL6 mice	Depression↓	Neuronal loss↓	MD2
Comim et al. [40]	Imipramine (10 mg/kg, ip)	CLP, Wistar rats	Depression↓	Corticosterone↓, ACTH↓, BDNF↑	HPA axis
Ranjbaran et al. [46]	Mesenchymal stem cells and their conditioned medium (1 × 10^6^ cells, ip)	CLP, Wistar rats	Anxiety↓	Inflammation↓, 5-HT2A receptors↓, 5-HT1A receptors↑	Serotonergic pathway
Kitagawa et al. [47]	Sulfasalazine (100 mg/kg, ip)	LPS, C57BL/6 mice	Depression↓	Glutamate release↓	System x_c_^−^
Cassol et al. [78]	Dexamethasone (0.2 mg/kg, ip)	CLP, Wistar rats	Depression↓	Corticosterone↓, ACTH↓	HPA axis
Petronilho et al. [79]	Guanosine (8 mg/kg, ip)	CLP, Wistar rats	Depression↓	Oxidative stress parameters↓	–
Leite et al [80]	Nicotine (0.1 mg/kg, subcutaneous)	CLP, Wistar rats	Anxiety↓	–	–
Ozcan et al. [81]	IgG (250 mg/kg, iv); IgGAM (250 mg/kg, iv)	CLP, Wistar albino rats	Anxiety and depression↓	–	–
Chen et al. [82]	NU9056 (5 mg/kg, ip)	LPS, C57BL/6 J mice	Anxiety and depression↓	BBB disruption↓, microglia activation↓, inflammatory markers↓, gut dysbiosis↓	NLRP3 inflammasome
Zhang et al. [83]	(R)-Ketamine (10 mg/kg, ip)	LPS, C57BL/6 mice	Delirium↓	Levels of inflammatory cytokines↓	–

## COVID-19 related psychiatric disorder

Psychiatric disorder can occur during both the acute phase and recovery period following a coronavirus disease 2019 (COVID-19) infection. This disorder includes anxiety, depression, PTSD, mania, delirium, and insomnia [84]. The main mechanism is direct neuroinvasion and neuroinflammation [85]. Unlike common sepsis, the development of COVID-19-related psychiatric impairment is influenced by pandemic-related stress and social isolation [84]. Managing and preventing COVID-19-related psychiatric disorder have become more challenging in the pandemic context. It has been demonstrated that supplementing with omega-3 can effectively decrease the likelihood of experiencing psychiatric consequence, such as depression, anxiety, and insomnia, after contracting COVID-19 [86]. In addition, Strict social isolation measures further hinder the implementation of the ABCDEF bundle, which makes it more difficult to control delirium [87]. Similarly, non-drug therapy is the primary approach to treating COVID-19-related psychiatric impairment at the moment. For example, narrative exposure therapy (NET) has been found to significantly ameliorate PTSD symptom after discharge [88]. Additionally, computerized cognitive behavioral therapy (cCBT) and progressive muscle relaxation training can greatly improve depression, anxiety, and sleep quality in COVID-19 patients [89, 90]. Furthermore, effective screening, early identification, and appropriate treatment are practical measures for managing delirium [91].

## Conclusion

**Fig. 1:**
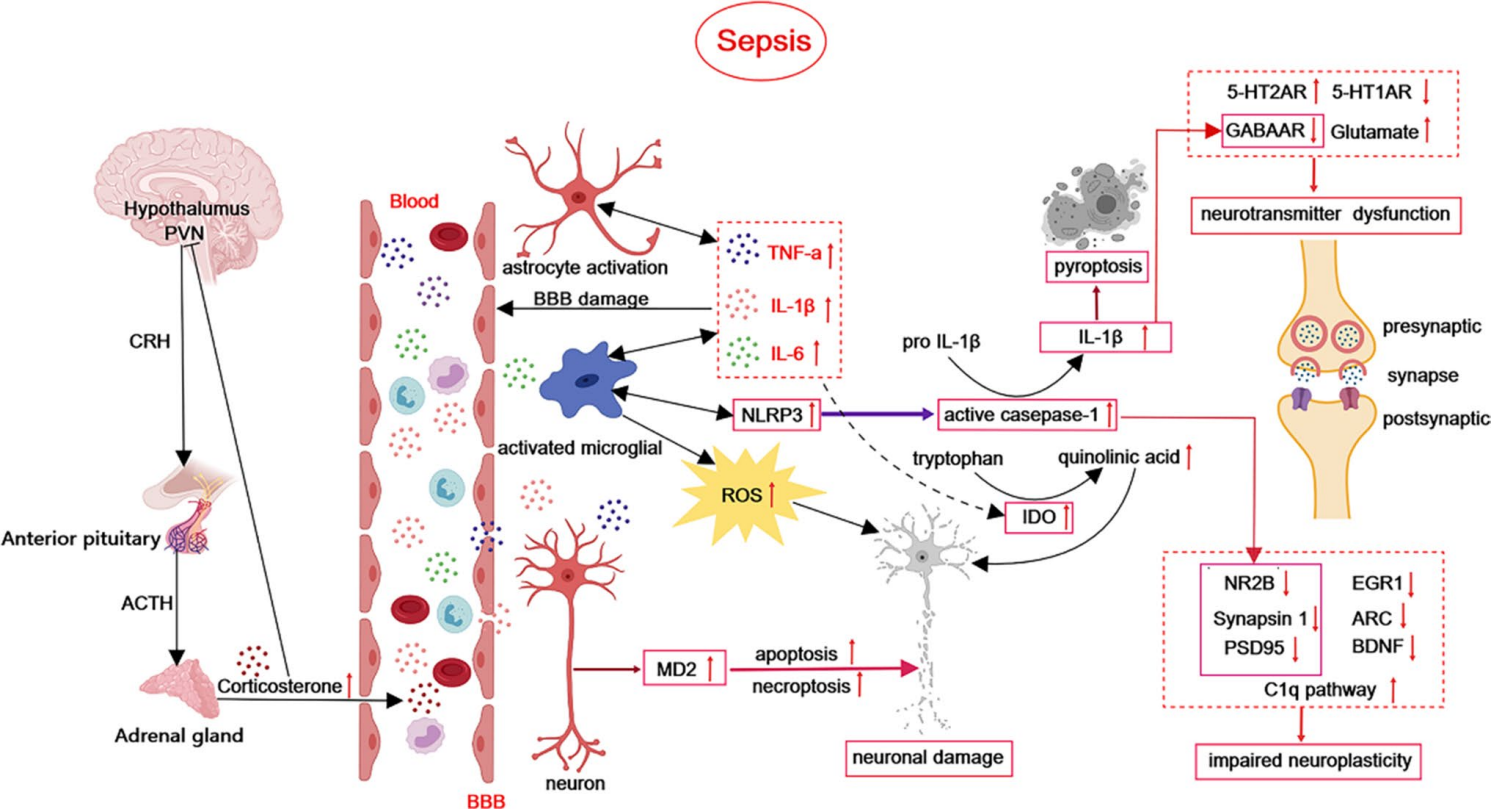
Overview of the pathophysiology of post-sepsis psychiatric disorder. During sepsis, the HPA axis becomes activated, resulting in increased release of glucocorticoids. Peripheral proinflammatory cytokines (TNF-α, IL-1β, and IL-6) enter the brain and induce neuroinflammation. Additionally, microglia and astrocyte activation leads to the release of proinflammatory cytokines, which disrupt the BBB. NLRP3 inflammasome activation promotes pyroptosis, and the increased production of IDO, ROS, and MD2 collectively leads to neuronal damage. Moreover, abnormal expression of plasticity-related proteins and genes results in impaired neuroplasticity. The dysfunction of the neurotransmitter system is a consequence of the atypical expression of neurotransmitters and their receptors

Psychiatric disorder secondary to sepsis has a significant impact on the quality of life of sepsis survivors. The pathogenesis of this condition includes BBB disruption, neuroinflammation, oxidative stress, PCD, impaired neuroplasticity, neurotransmitter dysfunction, and overactivation of the HPA axis (Fig. 1). The incidence of post-sepsis psychiatric disorder may be reduced by avoiding related risk factors. Currently, there is a dearth of effective treatments for psychiatric disorder following sepsis, and additional basic research is needed to identify potential therapeutic drugs targeting the underlying pathophysiology of the condition. Furthermore, non-pharmacological therapy should be considered as a viable treatment option in clinical practice. Additional research is required on the mechanism, prevention, and treatment of psychiatric impairment that arises after sepsis.

## Data Availability

All data analysed are included in this article.

## Abbreviations

*CMI*: 3-[(4-Chlorophenyl)selany1]-1-methyl-1H-indole
*Tat-CIRP*: Tat–cold-inducible RNA binding protein
*IgG*: Immunoglobulin G
*IgGAM*: Immunoglobulins enriched with IgA and IgM
*IgM*: Immunoglobulin M
*IgA*: Immunoglobulin A
*ip*: Intraperitoneal
*iv*: Intravenous
*BBB*: Blood–brain barrier
*CLP*: Cecal ligation and puncture
*NLRP3*: NOD-like receptor pyrin domain-containing protein 3
*NF-κB*: Nuclear factor-kappa B
*EGR1*: Early growth response 1
*LPS*: Lipopolysaccharide
*MD2*: Myelocyte differentiation factor 2
*IL-1β*: Interleukin-1beta
*ACTH*: Adrenocorticotropic hormone
*BDNF*: Brain-derived neurotrophic factor
*HPA*: Hypothalamic–pituitary–adrenal
*5-HT*: 5-Hydroxytryptamine
*TNF-alpha*: Tumor necrosis factor-α
*IFN-γ*: Interferon-gamma
*PVN*: Paraventricular nucleus
*CRH*: Corticotropin-releasing hormone
*ROS*: Reactive oxygen species
*iNos*: Inducible nitric oxide synthase
*PCD*: Programmed cell death
*NMDA*: N-methyl-D-aspartate
*Tspo*: Translocator protein
*GR*: Glucocorticoid receptor
*IDO*: Indoleamine 2,3-dioxygenase
*5-HT2AR*: 5-Hydroxytryptamine 2A receptor
*5-HT1AR*: 5-Hydroxytryptamine 1A receptor
*GABAAR*: Gamma amino butyric acid type A receptor
*NR2B*: N-methyl-D-aspartate receptor subunit 2B
*PSD-95*: Postsynaptic density protein 95
*EGR1*: Early growth response 1
*ARC*: Activity-regulated cytoskeletal-associated protein
*BAI*: Beck Anxiety Inventory
*SAS*: Self-Rating Anxiety Scale
*HADS-A*: Hospital Anxiety and Depression Scale-Anxiety
*MDI*: Major depression inventory
*BDI-II*: Beck Depression Inventory-II
*SDS*: Self Rating Depression Scale
*PHQ-9*: Patient Health Questionnaire -9
*HADS-D*: Hospital Anxiety and Depression Scale-Depression
*PCL-5*: PTSD checklist for DSM-5
*PTSS-10*: Posttraumatic symptom scale-10
*CAPS-5*: Clinician Administered PTSD Scale for DSM-5
*IES-R*: Impact of Events Scale–Revised
*PTSD*: Post-traumatic Stress Disorder
*DSM*: Diagnostic and Statistical Manual of Mental Disorders
*HRQOL*: Health-related quality of life
*IES-6*: Impact of Event Scale-6
*SCCM*: Society of Critical Care Medicine
*ICBT*: Internet-based Cognitive Behavioral Therapy
*COVID-19*: Coronavirus disease 2019
*cCBT*: Computerized cognitive behavioral therapy
*NET*: Narrative exposure therapy
*SAD*: Sepsis-associated delirium
*CAM-ICU*: Confusion Assessment Method for the Intensive Care Unit
*ICDSC*: Intensive Care Delirium Screening Checklist
*ABCDEF*: Assess, prevent, and manage pain; Both spontaneous awakening and breathing trials; Choice of Analgesia and Sedation; Delirium assess, prevent, and manage; Early Mobility and Exercise; Family engagement/empowerment
